# Human cytomegalovirus infection and cognitive decline: insights from population and experimental studies

**DOI:** 10.3389/fnagi.2025.1757461

**Published:** 2026-01-20

**Authors:** Koon Chu Yaiw, Isidre A. Ferrer, Cecilia Söderberg-Nauclér, Dorota Religa

**Affiliations:** 1 Department of Neurobiology, Care Sciences and Society (NVS), Karolinska Institutet, Stockholm, Sweden; 2 University of Barcelona, Barcelona, Spain; 3 Cell and Molecular Immunology, Karolinska University Hospital, Solna, Sweden; 4 Immunovirology, University of Turku, Turku, Finland; 5 Deparmtent of Neurology, Karolinska University Hospital, Stockholm, Sweden; 6 Department of Biosciences, InFLAMES Research Flagship Center, MediCity, University of Turku, Turku, Finland

**Keywords:** amyloid-beta, cognitive deficit, GSK-3 beta, Herp (HERPUD1), human cytomegalovirus, immediate-early protein, presenilin, tau

## Abstract

*Human cytomegalovirus (HCMV)*, a ubiquitous DNA betaherpesvirus, is capable of persistent infection and immunomodulation, particularly in immunocompromised and elderly hosts. Emerging evidence links HCMV to neurodegenerative diseases through its multifaceted immunomodulatory effects. This review summarizes key viral architectures and mechanisms, epidemiological trends, and experimental data supporting HCMV's role in cognitive decline. We advocate for targeted antiviral strategies and vaccine development to clarify its contribution to neurodegeneration.

## Introduction

### Human cytomegalovirus (HCMV): virological aspect

**Figure 1:**
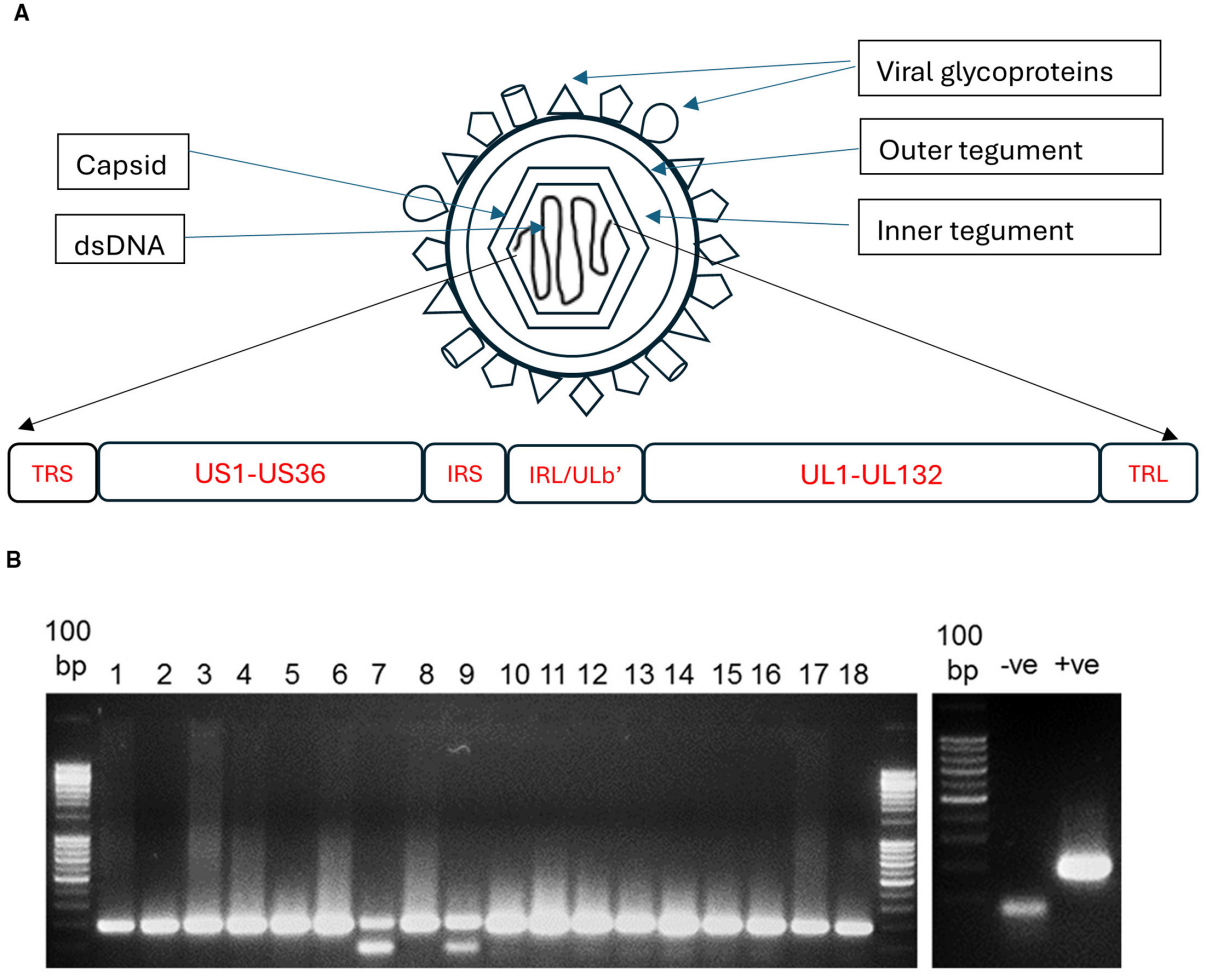
**(A)** Simplified graphics of human cytomegalovirus (HCMV) viral particle and its genomic organization. The HCMV genome is classified as an E-type structure, comprising unique long (UL) and unique short (US) regions flanked by terminal (TR) and internal (IR) repeats, which contain cleavage and packaging signals and enable genome isomerization. The UL133–UL138 locus, located within the ULb region (UL133–UL150), is retained in clinical and low-passage strains but frequently lost in laboratory-adapted viruses. Annotation of this region remains provisional. TRS, terminal repeat short. **(B)** Detection of HCMV in Alzheimer's disease brain tissue. **Left panel:** Amplification of the HCMV immediate-early (IE) gene from formalin-fixed paraffin-embedded (FFPE) sections of 18 Alzheimer's disease (AD) brains, demonstrating 100% positivity (18/18). **Right panel:** Positive (+ve) and negative (−ve) controls.

Human cytomegalovirus (HCMV), also known as human herpesvirus 5 (HHV-5), is a large, enveloped DNA virus within the *Betaherpesvirinae* subfamily of the *Herpesviridae* family. Its ~130 nm icosahedral capsid encloses a linear, double-stranded DNA genome of 235–250 kb (Suárez et al., 2019), organized into unique long (UL) and unique short (US) regions flanked by terminal and internal inverted repeats (Figure 1A). This complex architecture supports the expression of ~170 canonical and ~750 non-canonical open reading frames (ORFs; Stern-Ginossar et al., 2012), along with four large non-coding RNAs (Gatherer et al., 2011), two oriLyt RNAs (Kagele et al., 2012), and at least 24 microRNAs (Meshesha, 2012). These non-coding elements regulate both viral and host gene expression, contributing to immune evasion, latency, and pathogenesis across a range of disease contexts (Zeng et al., 2023; Ye et al., 2020).

Ribosome profiling in HCMV strain Merlin-infected fibroblasts identified 751 translated ORFs, underscoring the virus's expansive coding potential (Stern-Ginossar et al., 2012). Notably, over 70% of the genome encodes proteins involved in cell tropism and immune modulation, which are largely dispensable for replication *in vitro* (Dunn et al., 2003).

The capsid is composed of four essential structural proteins: the major capsid protein (UL86), minor capsid protein (UL46), minor capsid-binding protein (UL85), and the smallest capsid protein (UL48A), all required for virion assembly and replication (Mocarski et al., 2013). Surrounding the capsid, the tegument layer contains 38 proteins that mediate viral entry, gene regulation, assembly, and immune evasion (Kalejta, 2008). Among these, pp65 (UL83) is the most abundant and a key component of capsidless dense bodies—non-infectious particles lacking capsids and genomes—which can constitute up to 50% of extracellular particles produced by HCMV infected cells and contribute to immune modulation (Kalejta, 2008).

HCMV acquires its envelope from the host cell membrane, incorporating viral glycoproteins that facilitate attachment, entry, and immune evasion. Up to 65 glycoprotein-encoding ORFs have been identified (Wang et al., 2021), with 29 distinct glycoproteins detected in purified virions and dense bodies (Varnum et al., 2004). While some mediate viral entry and egress, others, such as the G protein–coupled receptor (GPCR) homolog US28, modulate host signaling via β-arrestin, Gα12, and RhoA-dependent pathways, and are implicated in apoptosis, cell migration and oncomodulation (Vomaske et al., 2009).

### HCMV entry and early gene expression

HCMV employs multiple, cell type–specific entry pathways mediated by interactions between viral envelope glycoproteins and host cell receptors (Griffiths and Reeves, 2021). In fibroblasts, the trimeric complex gH/gL/gO engages platelet-derived growth factor receptor-α (PDGFR-α) and co-receptors to trigger membrane fusion. In epithelial and endothelial cells, the pentameric complex (gH/gL/UL128/UL130/UL131) binds to receptors such as CD147, neuropilin-2, and endothelin B receptor (unpublished data), facilitating pH-dependent uptake of the virus particle via endocytosis and macropinocytosis, which involves integrin and PDGFR alpha signaling. Recently, EphA2 was identified as a receptor that mediates gH/gL-dependent membrane fusion in glioblastoma cells (Dong et al., 2023). Additional glycoproteins, including gM/gN, gB, and the newly characterized gH/UL116 complex, also contribute to host cell engagement (Shang and Li, 2024; Vezzani et al., 2021).

Following entry into permissive cells, HCMV initiates a temporally regulated gene expression cascade. Immediate-early (IE) genes are transcribed first in the nucleus, setting the stage for early (E) gene expression required for viral DNA replication, and finally late (L) genes are expressed encoding structural components required for virion assembly within the cytoplasmic viral assembly compartment. The major immediate-early genes, UL122 and UL123, encode IE2 and IE1 proteins, respectively, are central transcriptional regulators of this cascade and play pivotal roles in antagonizing host immune responses and establishing a permissive environment for replication (Adamson and Nevels, 2020).

### Clinical importance of HCMV

HCMV is a globally prevalent pathogen, with seropositivity ranging from 45% to nearly 100%, depending on demographic and socioeconomic factors (Fowler et al., 2022). While typically asymptomatic in immunocompetent hosts, HCMV establishes lifelong latency and persistence and poses serious risks in immunocompromised individuals. It is the leading cause of congenital infection and a major opportunistic pathogen in transplant recipients and AIDS patients (Mocarski et al., 2013).

Transmission occurs via bodily fluids, vertical transfer, transfusion, and transplantation. Its reproductive number (~1.7–2.4; Colugnati et al., 2007) suggests relatively low contagiousness. A substantial portion of the HCMV genome is dedicated to inflammation and immune evasion, raising interest in its potential role in chronic diseases, especially chronic inflammatory diseases, cardiovascular and autoimmune disorders, cancer, and neurodegeneration.

This review focuses on the virological features of HCMV that may contribute to cognitive impairment, drawing on epidemiological, experimental, and mechanistic evidence. Recent reviews have further explored its association with neurological disorders (Sanami et al., 2024).

## HCMV and cognitive decline: epidemiological and experimental evidence

### Epidemiological associations

The association between HCMV infection and cognitive impairment has been explored across multiple large-scale studies, though findings remain heterogeneous. In the Sacramento Area Latino Study on Aging (SALSA), a prospective cohort of 1,204 older Mexican Americans (mean age 70.3 ± 6.8), higher HCMV IgG levels—but not HSV-1—were significantly associated with accelerated cognitive decline over four years, independent of age, sex, education, income, and comorbidities (Aiello et al., 2006). Similarly, in a biracial cohort of 849 individuals (mean age 78.6 ± 7.2), HCMV seropositivity was linked to a 2.15-fold increased risk of Alzheimer's disease (AD) and faster global cognitive decline, independent of HSV-1 status, APOE ε4 genotype, and vascular risk factors (Barnes et al., 2015).

In a broader U.S. cohort of 5,617 adults (mean age ~75), Stebbins et al. reported a trend toward reduced cognitive performance in HCMV-seropositive individuals, though the association was not statistically significant after adjustment for confounders (Stebbins et al., 2021). The Monongahela-Youghiogheny Healthy Aging Team (MYHAT) study, which followed 1,022 participants aged ≥65 (mean age 77.5 ± 7.5) over five years, found that IgG levels for HCMV, HSV-2, and *Toxoplasma gondii*—but not HSV-1—were significantly associated with temporal cognitive decline (Nimgaonkar et al., 2016).

Additional evidence comes from a Japanese cohort of 494 individuals aged >85, where HCMV seropositivity predicted cognitive decline specifically in those with carotid atherosclerosis (Kawasaki et al., 2016). A population-based study also reported increased odds of all-cause dementia (OR = 1.9) and vascular dementia (VaD) (OR = 2.9) in HCMV-seropositive individuals (Lee et al., 2020).

A recent meta-analysis of seven studies (*n* = 6,772) confirmed a significant association between HCMV infection and AD risk, particularly in East Asian populations (OR = 2.39; 95% CI: 1.63–3.50), cohort studies (OR = 1.99; 95% CI: 1.35–2.94), and studies with confounder adjustment (OR = 2.05; 95% CI: 1.52–2.77) (Ji et al., 2024). These findings suggest that HCMV may contribute to cognitive decline through mechanisms involving chronic inflammation, vascular dysfunction, and host susceptibility.

### Insights from animal models

Animal studies have also provided mechanistic insights into how cytomegalovirus infection may contribute to neurodegeneration. *In vitro*, murine CMV (MCMV) infection induces tau pathology in mouse fibroblasts and rat neuronal cells, dependent on late viral gene expression but independent of glycogen synthase kinase 3β (GSK3β) activity—suggesting an alternative pathway for tau phosphorylation (Mody et al., 2023).

*In vivo*, repeated systemic MCMV infection in mice has been shown to elevate neuroinflammatory markers, disrupt mitochondrial function, increase oxidative stress, and impair cognitive performance (Harrison et al., 2023). Consistent with these findings, a recent preprint using 3xTg-AD transgenic mice demonstrated that systemic MCMV infection accelerates hallmark features of Alzheimer's disease (AD), including cognitive decline, tau hyperphosphorylation, and synaptic loss in the hippocampus (Marsden et al., 2025).

Further supporting a causal link, Liu et al. (2024) engineered HCMV IE2 transgenic mice with hippocampus-specific expression of the IE2 protein. These mice exhibited elevated levels of amyloid precursor protein (APP) and phosphorylated tau (p-Tau), recapitulating key aspects of AD-like pathology (Liu et al., 2024).

### Laboratory evidence and potential neurophysiological mechanisms linking HCMV to cognitive decline

Postmortem and *in vitro* studies further implicate HCMV in neurodegenerative processes. In a PCR-based analysis, HCMV DNA was detected in 93% of brain specimens from patients with vascular dementia (VaD), compared to 34% of age-matched controls (Lin et al., 2202). The high prevalence of HCMV in samples from patients with dementia, were corroborated by our own analyses of brain tissue samples from 18 individuals with Alzheimer's disease (AD) and 18 age-matched individuals classified as having “normal aging” brains. Using an in-house nested PCR assay targeting the HCMV immediate-early (IE) gene (Söderberg et al., 1993), along with our optimized DNA extraction method, we successfully detected HCMV in all 18 AD samples (100%; Figure 1B), but viral presence was also evident in 17 out of 18 (94.7%) of the normal aging samples. The authenticity of the amplicons for HCMV was confirmed by Sanger sequencing (data not shown).

In AD patients, HCMV seropositivity has been associated with increased neurofibrillary tangle (NFT) burden and elevated interferon-γ levels in cerebrospinal fluid (CSF)—a cytokine detected only in seropositive individuals (Lurain et al., 2013). HCMV-seropositive subjects also exhibited a higher proportion of senescent T cells (CD4^+^ or CD8^+^CD28^−^CD57^+^), which were marginally associated with AD pathology and amyloid-β levels. Notably, infection of human foreskin fibroblasts (HFFs) with clinical HCMV strains induced amyloid-β expression, whereas HSV-1 infection did not—highlighting an HCMV-specific effect (Lurain et al., 2013). Recent studies using human cerebral organoids have further reinforced these findings. HCMV infection (MOI = 2) accelerated production of Aβ42 and phosphorylated tau (pTau-212), and induced neuronal death (Readhead et al., 2025). Transcriptomic profiling revealed HCMV-mediated downregulation of genes involved in cortical development and neuronal function, independent of viral load (O'Brien et al., 2022).

Although the accumulation of Aβ and tau proteins is a defining feature of AD, the mechanisms underlying their aggregation remain only partially understood. HCMV encodes more than 200 proteins, among which the IE proteins IE1-p72 and IE2-p86 are essential for viral replication and contribute to the pathogenesis of multiple diseases. HCMV's capacity to establish lifelong latency, undergo periodic reactivation, secrete proinflammatory cytokines, and exhibit increasing seropositivity with age is associated with pathological outcomes such as atherosclerosis and certain cancers. Through its sophisticated modulation of host cellular and immune pathways, HCMV may also promote cognitive decline via mechanisms that directly or indirectly contribute to AD-related pathology. These mechanisms include effects on glial cell function (particularly microglia and astrocytes), alterations in local brain cytokine profiles, disruption of blood–brain barrier integrity, and dysregulation of Aβ and tau metabolism, presenilin (PS), glycogen synthase kinase (GSK)-3β, and HERPUD1 (a homocysteine-responsive, ER stress-inducible protein). Collectively, these disturbances can impair synaptic transmission and plasticity, disrupt hippocampal and prefrontal network function, and destabilize inflammatory cytokine balance within the brain.

**Figure 2:**
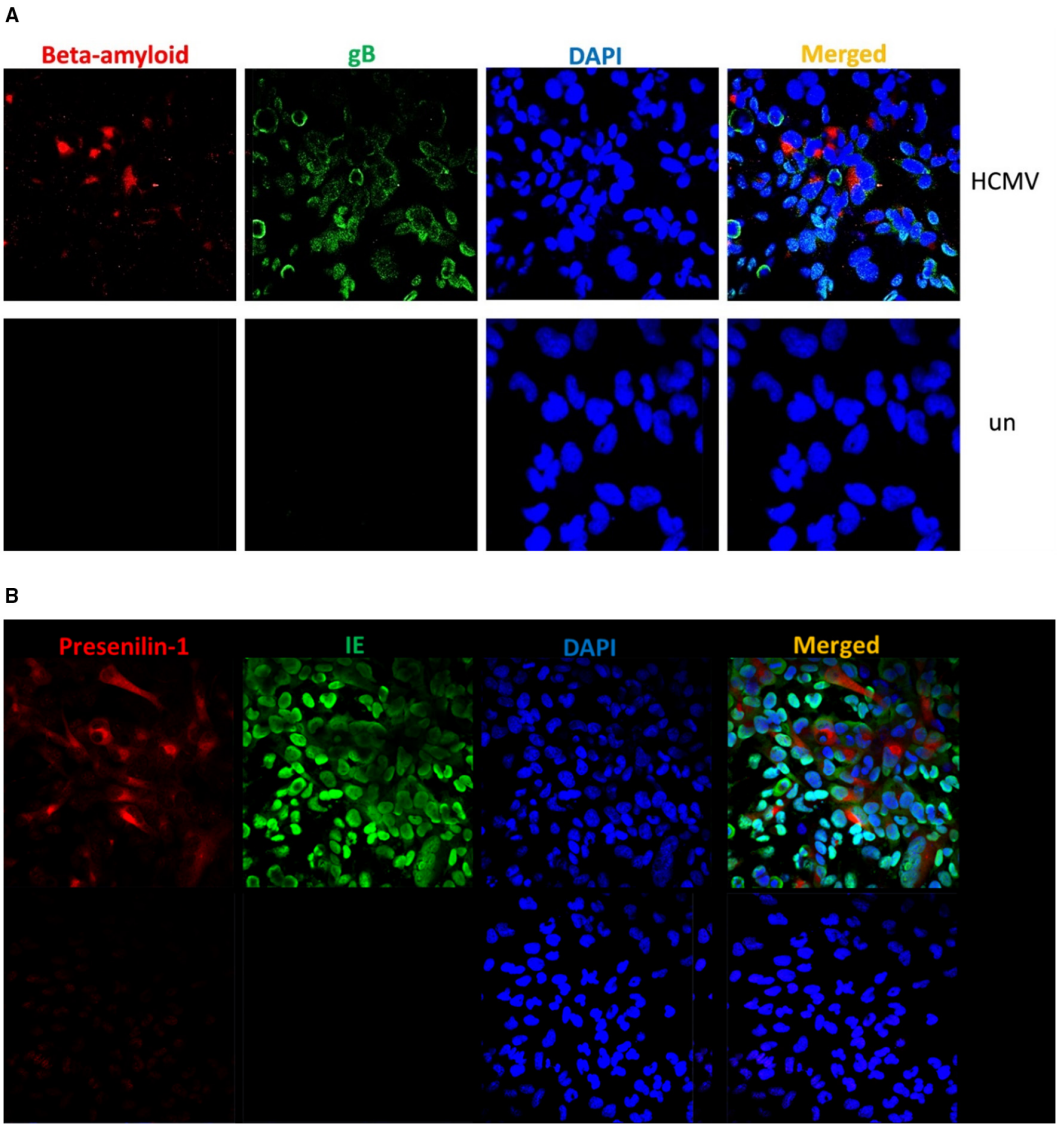
**(A)** HCMV induces amyloid-β accumulation in U251MG cells. Immunofluorescence staining of U251MG glioblastoma cells at 3 days post-HCMV infection (*n* = 2). Amyloid-β is shown in red, HCMV glycoprotein B (gB) in green, and nuclei in blue (DAPI). The observed co-localization indicates HCMV-induced accumulation of amyloid-β, suggesting a potential mechanistic link to AD pathology. **(B)** HCMV infection upregulates Presenilin-1 expression in U251MG cells. Immunofluorescence staining of U251MG glioblastoma cells at 3 days post-infection with HCMV (*n* = 2). Viral immediate-early (IE) protein is shown in green, and Presenilin-1 (PS-1) in red. Co-localization indicates HCMV-induced upregulation of PS-1, suggesting a potential mechanistic link to AD-related pathology.

To further explore the potential link between HCMV and AD, we infected U251MG glioblastoma cells with HCMV and performed immunofluorescence staining at three days post-infection. Cells were stained for either Aβ or presenilin-1 (PS-1; Figures 2A, B), in combination with viral immediate-early (IE) protein or glycoprotein B (gB). Our results revealed that HCMV infection induces the accumulation of both Aβ and PS-1, suggesting a direct contribution to AD-related pathology.

This association is further supported by the upregulation of HERPUD1 transcripts following HCMV infection, as revealed by RT^2^ Profiler PCR array analysis (Supplementary 1) in our pilot study investigating molecular pathways targeted by HCMV in endothelial cells. Notably, HERPUD1/Herp protein overexpression has been implicated in promoting Aβ accumulation (Sai et al., 2002). This effect was significantly attenuated by BQ788, a selective antagonist of the endothelin B receptor (ETBR; Yaiw et al., 2015; Landázuri et al., 2021). Notably, we recently identified ETBR as a critical mediator of HCMV pentamer dependent entry and replication in both endothelial, epithelial cells and macrophages (unpublished data) Consistent with this, treatment with BQ788 restored HERPUD1 transcript levels to nearly baseline (Supplementary 1), reinforcing the role of HCMV-mediated signaling in AD pathogenesis. Future studies in neuronal cells are warranted to validate these findings.

Mechanistically, HCMV reactivation is triggered by oxidative stress, DNA damage, and inflammatory cytokines (Merchut-Maya et al., 2022; Söderberg-Nauclér et al., 1997) factors that also promote lytic gene expression and viral dissemination under immunosuppressive conditions (Heald-Sargent et al., 2020). In fact, HCMV itself induces oxidative stress and inflammation in infected cells—processes that are all implicated in AD pathogenesis. Given the pivotal role of GSK-3β in linking senile plaques to neurofibrillary tangles, we investigated whether HCMV infection modulates GSK-3β activity in human umbilical vein endothelial cells (HUVECs). Proteome profiler array analysis revealed that HCMV infection indeed induces phosphorylation of GSK-3β (Supplementary 2), further supporting its role in virus-host interactions relevant to AD pathogenesis.

Importantly, HCMV seropositivity increases with age and is closely linked to immunosenescence. Latent infection exerts a profound influence on the aging immune system, potentially compounding the risk of cognitive decline in older adults (Sansoni et al., 2014).

### Discussion and conclusion

Evidence from prospective epidemiological studies, animal models, and laboratory investigations increasingly implicates HCMV in the pathogenesis of cognitive impairment and even dementia, in particular AD.

The presence of HCMV in healthy brains may represent an epiphenomenon—modifying risk rather than acting as a single causal factor. Its influence on cognitive decline is likely shaped by additional predisposing conditions, including viral load, regional susceptibility within the brain, vascular pathology, APOE4 genotype, and aging.

Further insight into how HCMV modulates brain function, cytokine signaling, and cognitive outcomes will require integrative experimental approaches. Studies using transgenic HCMV–AD animal models and brain organoids, combined with electrophysiological recordings or calcium imaging, as well as investigations into HCMV's effects on the blood–brain barrier and glial cell responses, will be essential for elucidating the mechanisms by which HCMV contributes to neurodegenerative processes.

While a causal role for HCMV in neurodegeneration remains unproven, future studies—particularly those leveraging antiviral therapies or vaccines aimed at preventing AD and vascular dementia—may clarify whether the virus functions as an etiological contributor. Additional approaches, including probiotics or fecal microbiota transplantation that influence HCMV latency and reactivation, also warrant close investigation as potential strategies to mitigate cognitive decline in susceptible populations.
