# Supplementary material for: Human cytomegalovirus infection and cognitive decline: insights from population and experimental studies
Source: Front Aging Neurosci. 2026 Jan 20;17:1757461. doi: 10.3389/fnagi.2025.1757461 (PMC12865707; doi:10.3389/fnagi.2025.1757461)
Supplement: Supplementary file 1 [file Table_1.docx]

**Supplementary figures**

*
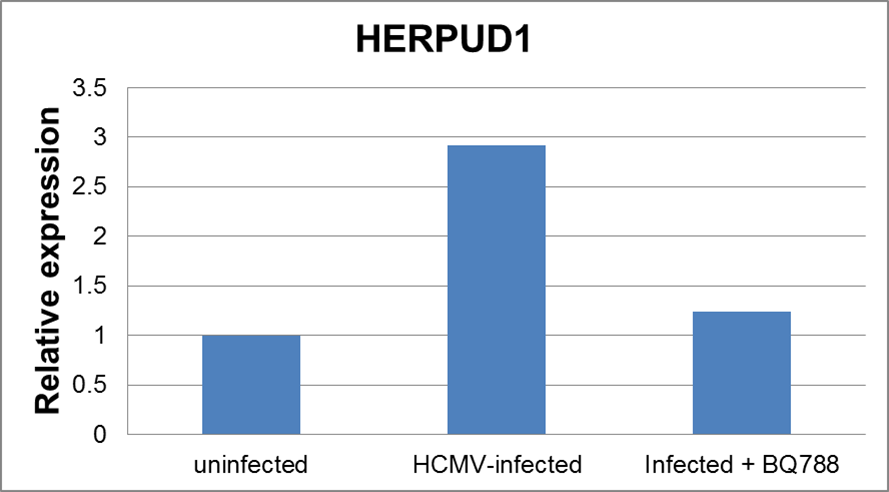
*

**Suppl.1 | Upregulation of HERPUD1 in HUVECs following HCMV infection is mitigated by BQ788 treatment.**
Expression of HERPUD1 transcripts was assessed in a pilot study using human umbilical vein endothelial cells (HUVECs) post-HCMV infection using RT² Profiler PCR array. HCMV infection significantly upregulated HERPUD1 expression, which was effectively mitigated by BQ788, a selective endothelin B receptor (ETBR) antagonist, indicating ETBR-mediated regulation of HERPUD1 in response to viral infection.


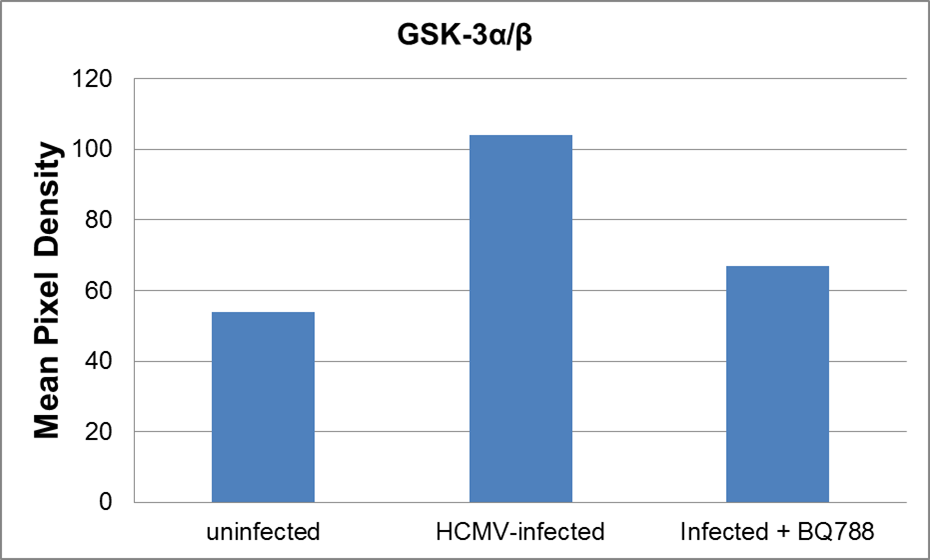


**Suppl.2 | HCMV-induced activation of GSK-3β in HUVECs is attenuated by BQ788 treatment.**
Human umbilical vein endothelial cells (HUVECs) were infected with HCMV and analyzed for GSK-3β activation via proteome profiler array. HCMV infection led to increased phosphorylation of GSK-3β, indicative of kinase activation. This effect was mitigated by BQ788, a selective endothelin B receptor (ETBR) antagonist, highlighting the role of ETBR signaling in HCMV-mediated modulation of GSK-3β.
